# Mortality rate trends in patients diagnosed with schizophrenia or bipolar disorder: a nationwide study with 20 years of follow-up

**DOI:** 10.1186/s40345-018-0140-x

**Published:** 2019-03-01

**Authors:** Line Hosbond Lomholt, Diana Vincens Andersen, Christina Sejrsgaard-Jacobsen, Cagla Margit Øzdemir, Claus Graff, Ole Schjerning, Svend Eggert Jensen, Sune Puggard Vogt Straszek, Rasmus W. Licht, Simon Grøntved, René Ernst Nielsen

**Affiliations:** 1 Department of Clinical Medicine, Aalborg University, Aalborg, Denmark; 2 Department of Health Science and Technology, Aalborg University, Aalborg, Denmark; 3 Department of Psychiatry, Aalborg University Hospital, Mølleparkvej 10, 9000 Aalborg, Denmark

**Keywords:** Bipolar disorder, Schizophrenia, Epidemiology, Mortality

## Abstract

**Background:**

Patients with severe mental illness (SMI) have a reduced life expectancy of one to two decades as compared to the general population, with most years of life lost due to somatic diseases. Most previous studies on disorders constituting SMI, e.g. schizophrenia and bipolar disorder, have investigated the disorders separately and hence not compared the disorders in terms of mortality rates relative to the background population.

**Methods:**

A register-based cohort study including the entire Danish population comparing mortality rates relative to the background population, controlling for age and sex, i.e. standardized mortality ratios (SMRs) in patients diagnosed with schizophrenia with those in patients diagnosed with bipolar disorder, during the study period from 1995 to 2014.

**Results:**

The SMR of patients with SMI was significantly higher than one for each calendar year in the study period with an overall SMR of 4.58, 95% CI (4.48–4.69) in patients diagnosed with schizophrenia (n = 38,500) and of 2.57 (95% CI 2.49–2.65) in patients diagnosed with bipolar disorder (n = 23,092). When investigating time trends in SMR for schizophrenia and for bipolar disorder, respectively, an increase in SMR over time was shown with a mean increase of 0.03 per year for schizophrenia and 0.02 for bipolar disorder (p < 0.01 for both disorders). The ratio between SMR for schizophrenia and SMR for bipolar disorder for each calendar year over the study period was constant (p = 0.756).

**Conclusions:**

Increasing SMRs over the last 20 years were found for both patients diagnosed with bipolar disorder and patients diagnosed with schizophrenia. Despite clear differences between the two disorders regarding SMRs, the increases in SMR over time were similar, which could suggest similar underlying factors influencing mortality rates in both disorders.

## Background

Patients diagnosed with bipolar disorder and patients diagnosed with schizophrenia, constituting a group of patients with severe mental illness (SMI), have a reduced life expectancy of one to two decades as compared to the background population (Nordentoft et al. 2013). There is a large increased relative risk of suicide or accidents as the cause of death, but most years of life lost are attributable to somatic disorders, similar to the findings in the general population (Nordentoft et al. 2013). Specialized treatment interventions have been shown to be effective in reducing the psychiatric outcomes as relapse and hospitalization (Kessing et al. 2013; Correll et al. 2018), but so far outcome data on mortality and somatic morbidity have not been reported. Patients with severe mental illness have an increased rate of poor life style such as lack of exercise, poor diet, increased occurrence of smoking and obesity, resulting in increased rates of chronic somatic diseases leading to excess mortality rates (Mitchell et al. 2015; Vancampfort et al. 2015, 2016, 2017). In a recent study by Speyer et al. no effect of care coordination and lifestyle coaching was seen on the primary outcome of 10-year risk of cardiovascular disease when compared to treatment as usual (Speyer et al. 2016). In the study by Kugathasan et al. investigating long-term outcome after myocardial infarction in patients with schizophrenia, no difference in short-term outcome compared to patients experiencing myocardial infarction but not diagnosed with schizophrenia was shown, whereas an increased long term mortality was reported (Kugathasan et al. 2018). The study by Jakobsen et al. showed similar results and showed furthermore no difference in the in-hospital treatment between patients with SMI and the background population, whereas a lower percentage of patients with SMI received the recommended medical treatment during follow-up resulting in an increased risk of new adverse cardiac events (Jakobsen et al. 2017).

Most studies have reported life expectancy or mortality rates for psychiatric disorders separately and not investigated the mortality for several disorders to compare the disorders with each other. Several studies have shown an increasing mortality gap between patients diagnosed with schizophrenia, patients diagnosed with bipolar disorders and comparative non-psychiatric populations (Nielsen et al. 2013; Hayes et al. 2017). We have previously studied the time trends of standardized mortality ratios (SMRs), i.e. mortality relative to the background population controlling for age and gender, in patients diagnosed with bipolar disorder over the last 20 years (Staudt Hansen et al. 2018). In the current study, we aimed at investigating SMRs for each single year from 1995 to and including 2014 for both schizophrenia and bipolar disorder. Furthermore, we aimed at comparing the two disorders regarding time trends in the SMRs over the study period.

## Methods

### Design

A register-based cohort study including the entire Danish population.

### Study population

All patients registered with a schizophrenia or bipolar disorder diagnosis in the Danish Psychiatric Central Research Registry (DPCRR) from 1965 until the end of 2014, living in Denmark, alive and below the age of 65 years in the study period from 1995 to 2014 were defined as the severe mental illness population and included in the current study.

Included patients reaching the age of 65 years during the study period were censored at that time point. Schizophrenia was defined as an ICD-8 300.x diagnosis in the period 1965 to end of 1994 or and ICD-10 F20.x diagnosis in the period 1995 to end of 2014. Bipolar disorder was defined as an ICD-8 296.x diagnosis in the DPCRR in the period 1965 to the end of 1994 or an ICD-10 F30.x or F31.x diagnosis in the period 1995 to the end of 2014, resulting in the inclusion of both incident and prevalent cases in contact with hospital-based in- or out-patient treatment facilities. If patients were diagnosed with both bipolar disorder and schizophrenia, patients were analyzed according to the schizophrenia diagnosis.

### Registers utilized

Data on psychiatric contacts were retrieved from the DPCRR (Mors et al. 2011) and the Danish National Patient Register (Lynge et al. 2011).

Data on date of deaths of patients and background population were retrieved from the Danish Civil Registration System (Pedersen 2011).

All inhabitants of Denmark have a unique person identification number—civil person registration number (CPR)—that enables linkage between registers.

### Statistical analyses

The standardized mortality ratio (SMR) for the whole study period was calculated separately for patients diagnosed with schizophrenia and for patients diagnosed with bipolar disorder as the observed number of deaths among patients with either schizophrenia or bipolar disorder divided by the expected number of deaths based on the age and gender specific mortality rates of the general population (Naing 2000). Secondly, time trends of SMR during the 20-year follow-up period were assessed using random effects linear regression, with SE as random effect (Juliouset al. 2001), setting the significance level of the *p* value of the slope < 0.05, for each of the two diagnostic groups. Lastly, time trends of ratios of annual SMR for schizophrenia to annual SMR for bipolar disorder during the 20-year follow-up period were assessed using random effects linear regression, with SE as random effect (Julious et al. 2001), setting the significance level of the p-value of the slope < 0.05.

Data management and statistical analyses were performed in Stata 15 (StataCorp, College Station, TX, USA).

## Results

The total population consisted of 6,176,414 persons with 38,500 patients (23,625 male and 14,875 female) with a schizophrenia diagnosis and 23,092 (9510 male and 13,582 female) patients with a bipolar disorder diagnosis alive in the period 1995 to 2014.

The SMR of patients with SMI was significantly higher than one for each calendar year in the study period (Table 1), with an overall SMR of 4.58, 95% CI (4.48–4.69) in patients diagnosed with schizophrenia and 2.57 (95% CI 2.49–2.65) in patients diagnosed with bipolar disorder for the whole study period.

**Table 1:** Standardized mortality ratios in the study period for patients diagnosed with schizophrenia and patients diagnosed bipolar disorder, respectively

Year	Schizophrenia					Bipolar disorder				
	Obs deaths	Exp deaths	SMR	95% CI		Obs deaths	Exp deaths	SMR	95% CI	
1995	278	67.25	4.13	3.68	4.65	279	121.60	2.29	2.04	2.58
1996	265	68.95	3.84	3.41	4.34	299	115.51	2.59	2.31	2.90
1997	303	70.52	4.30	3.84	4.81	253	108.64	2.33	2.06	2.63
1998	309	71.51	4.32	3.86	4.83	243	104.42	2.33	2.05	2.64
1999	348	74.31	4.68	4.22	5.20	250	102.31	2.44	2.16	2.77
2000	316	75.20	4.20	3.76	4.69	212	97.53	2.17	1.90	2.49
2001	357	78.25	4.56	4.11	5.06	238	94.60	2.52	2.22	2.86
2002	370	81.58	4.54	4.10	5.02	252	91.85	2.74	2.42	3.10
2003	400	84.71	4.72	4.28	5.21	248	89.62	2.77	2.44	3.13
2004	409	86.25	4.74	4.30	5.22	241	87.22	2.76	2.44	3.14
2005	433	84.30	5.14	4.67	5.64	201	82.94	2.42	2.11	2.78
2006	428	89.40	4.79	4.35	5.26	236	84.30	2.80	2.46	3.18
2007	447	93.32	4.79	4.37	5.26	222	84.95	2.61	2.29	2.98
2008	408	90.16	4.53	4.11	4.99	245	79.95	3.06	2.70	3.47
2009	417	92.10	4.53	4.11	4.98	196	78.44	2.50	2.17	2.87
2010	419	92.46	4.53	4.12	4.99	195	75.31	2.59	2.25	2.98
2011	412	88.06	4.68	4.25	5.15	195	69.74	2.80	2.43	3.22
2012	410	86.48	4.74	4.30	5.22	176	66.87	2.63	2.27	3.05
2013	397	85.74	4.63	4.20	5.11	174	64.81	2.68	2.31	3.11
2014	419	85.37	4.91	4.46	5.40	180	64.07	2.81	2.43	3.25

*Obs deaths* observed number of deaths, *Exp deaths* expected number of deaths, *SMR* standardized mortality ratio, *CI* confidence interval

**Fig. 1:**
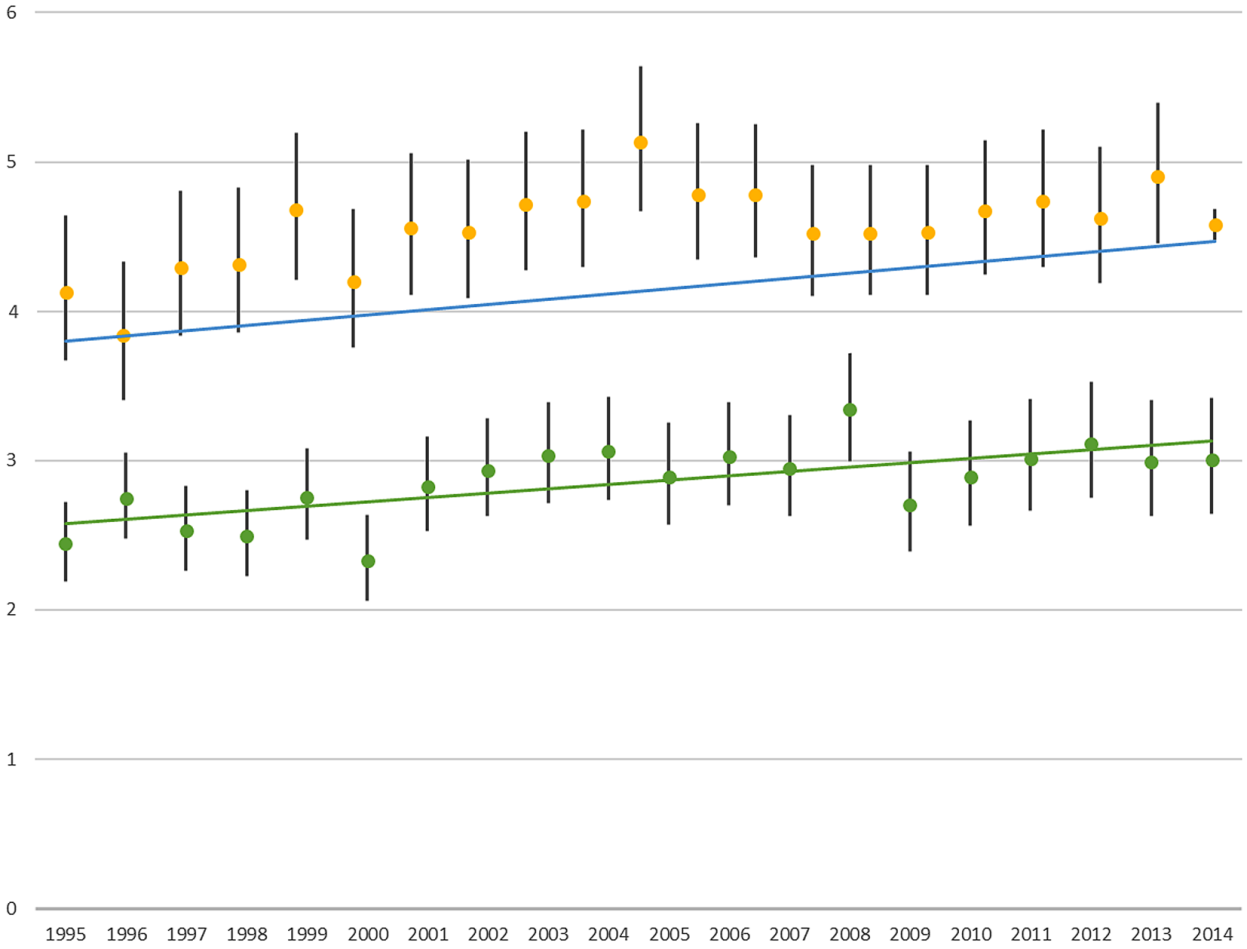
Standardized mortality ratios including 95% confidence intervals for patients diagnosed with schizophrenia (yellow points) and bipolar disorder (green points) compared to the general Danish population from 1995 to 2014. The dotted lines are fitted utilizing linear regression

When investigating time trends in SMRs for schizophrenia and bipolar disorder respectively an increase in SMR over time was shown with a mean increase of 0.03, p < 0.01, in SMR per year in patients diagnosed with schizophrenia and 0.02, p < 0.01, in SMR per year in patients diagnosed with bipolar disorder, as shown in Fig. 1.

**Fig. 2:**
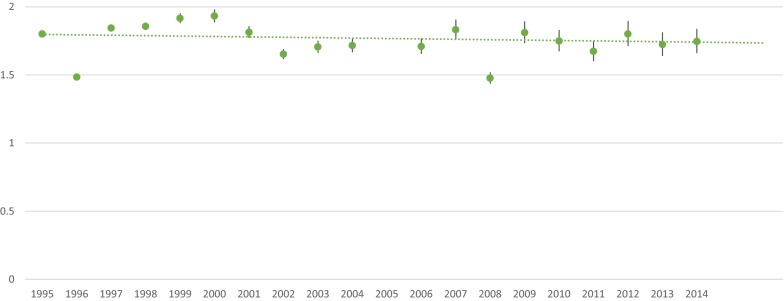
Ratio of standardized mortality ratio for schizophrenia divided by standardized mortality ratio for bipolar disorder. The dotted line is fitted utilizing linear regression

The ratio between annual SMRs for schizophrenia over the study period and annual SMRs for bipolar disorder over the study period was unchanged over the study period (p = 0.7560), as shown in Fig. 2.

## Discussion

In this nationwide study including more than 65,000 patients diagnosed with SMI below the age of 65, the overall mortality rate was more than four times higher for patients diagnosed with schizophrenia and more than two-and-a-half times higher for patients diagnosed with bipolar disorder than that of the general population, standardized for age and gender. Furthermore, the increase in annual SMRs over the study period was similar in the two groups diagnosed with SMI. Accordingly, the ratio between the annual SMRs for the two psychiatric populations was unchanged over time.

Similar to our findings, previous studies have shown increases in the relative mortality gap over time between the patients diagnosed with schizophrenia and bipolar disorder respectively and background populations (Hayes et al. 2015; Lee et al. 2018). Hayes et al. (2017) showed an increased mortality rate in both patients diagnosed with schizophrenia as well as in patients diagnosed with bipolar disorder, but did not find a two-fold difference in relative mortality rates as compared to the background population. Contrary to the currents study, Hayes et al. (2017) utilized several regression models to investigate time trends in mortality rates as well as to adjust to explanatory variables. Lastly, the study by Hayes et al. (2017) supported a general decrease in absolute mortality rates for all investigated, but a larger reduction in absolute mortality rates for the background population, resulting in an increasing mortality gap between patients diagnosed with bipolar disorder or schizophrenia as compared to the background population. Studies have also shown a general lowered life expectancy in patients diagnosed with SMI (Nordentoft et al. 2013), which might have been caused by poorer treatment of physical diseases (Laursen and Nordentoft 2011; Laursen et al. 2013; Kugathasan et al. 2018) and by higher prevalence of physical co-morbidities and more unhealthy lifestyle in patients with SMI as compared to the background population (Mitchell et al. 2015; Vancampfort et al. 2015, 2016, 2017). Poor lifestyle choices like low physical activity (Vancampfort et al. 2017), poor diet (Teasdale et al. 2017), and lack of smoking cessation (Mitchell et al. 2015) are still more prevalent in patients with SMI as compared to the general population. Also, cardiovascular risk factors, diabetes, dyslipidemia and obesity are more prevalent in patients with psychiatric disorders than in the background population, and the associations of these risk factors to psychiatric disorders are not significantly changed by treatment with antidepressants or antipsychotics, and these treatments themselves are associated to risk factors as well (Pérez-Piñar et al. 2016). Studies investigating the effects of life style interventions have been conducted, but have mainly shown disappointing results with lack of changes in risk profiles (Speyer et al. 2016; Ashdown-Franks et al. 2018).

Studies have raised concern regarding disparity in treatment of physical disease for patients with SMI, with a relatively low quality of care for patients with psychiatric diagnoses in general (Mitchell et al. 2014). In patients with SMI and also diagnosed with AIDS, a lowered compliance to medication was observed (Moore et al. 2012), and in patients with non-affective psychotic disorders a lowered prevalence of prescribed somatic medication and fewer consultations with a general practitioner has been observed (Swildens et al. 2016), in patients with SMI disparities in screening for cancer were observed as well as a higher rate of case-fatality, which was not reduced by adjusting for cancer stage and comorbidity (Howard et al. 2010; Manderbacka et al. 2017). In patients with SMI, a lowered rate of invasive cardiac procedures was observed as compared to the background population despite patients being admitted to hospital (Laursen et al. 2009). After treatment of somatic disorders an increased risk of early rehospitalization in patients with SMI was observed for cardiovascular, pulmonary, endocrinological and infectious diseases (Davydow et al. 2016), suggesting an unmet need for treatment or a poorer treatment response as compared to the background population. Patients with SMIs have a well-documented increased rate of mortality mainly as a consequence of somatic illnesses, even when adjusting for stage of illness (Laursen 2011; Wahlbeck et al. 2011). In accordance with these findings, a decreased proportion of patients with severe mental disorders were screened for breast cancer, cervical cancer or general somatic illness as compared to the background population (James et al. 2017; Thomas et al. 2018).

Data support a lack of knowledge of mental disorders as well as a more negative attitude towards patients with mental disorders in nursing staff in somatic care, as compared to nursing staff in mental health (Björkman et al. 2008). These negative attitudes, as well as the lack of integration between somatic and psychiatric treatment centers might result in the disparity in treatment of somatic disorders in patients with SMI, partially influencing mortality, and resulting in patients with SMI not gaining the same effects of reduced mortality as a result of improved treatment for somatic disease as the background population (Fleischhacker et al. 2008).

Our findings suggest that for schizophrenia and bipolar disorder there is a similar increase in mortality gap between the two disorders respectively and the background population. However, by utilizing SMRs as an outcome measure, which reports the relative mortality rates between a specified population and the background population, we were unable to confer if the increasing mortality ratio is a result of patients with SMI having an increased mortality rate absolutely over time or merely a less lowered mortality rate as compared to the background population. The similar time trends in SMR for patients diagnosed with schizophrenia and bipolar disorder could suggest similar underlying factors influencing mortality, although the design and model of analysis do not allow conclusions regarding causality.

### Strengths and limitations

In the current study, inclusion of both incident and prevalent cases was chosen to increase clinical relevance and generalizability of the results. Nationwide population-based register studies in Denmark benefit from several factors. First, reporting data to the Danish health care registers is mandatory for all in- or out-patients treated in a hospital-based service treatment. As a result, almost no patients are lost to follow-up. Secondly, in Denmark health care is without any direct cost for the individual, as healthcare is provided and paid as part of the Danish tax system. Consequently, results from Danish studies are not confined to patients with a certain socioeconomic status or patients from specific geographical regions of a larger country as some health insurance database studies might be. The schizophrenia diagnosis has been validated in the Danish healthcare registers (Uggerby et al. 2013), and generally the validity of psychiatric diagnoses is believed to be high (Mors et al. 2011), although no specific validation of the bipolar disorder diagnosis has been conducted. Due to the very long inclusion period, patients were included based on the ICD-8 (first part of the period) and on the ICD-10 (last part of the period). However, those patients diagnosed utilizing ICD-8 criteria might not have constituted the exact same patient group as those being diagnosed according to ICD-10.

The primary outcome of SMR carries some limitations. SMR is only adjusted for age and sex, resulting in lack of adjustment for other possible confounders. Utilizing other models would have allowed adjustment for more variables, which though could have obscured the overall effect of, e.g. life style and socio-economic status, which is systematically different in patients with SMI as compared to the background population. With the current model used, the true excess mortality of this disadvantaged patient group is reported. The current findings could be biased by including only patients assigned a register diagnosis. Thus, persons fulfilling criteria for schizophrenia or bipolar disorder, but not diagnosed in a psychiatric setting before death, could have biased the results conservatively.

## Conclusion

Increasing SMRs over the last 20 years were found for both patients diagnosed with bipolar disorder and patients diagnosed with schizophrenia. Despite clear differences between the two disorders regarding SMRs, the increases in SMR over time were similar, which could suggest similar underlying factors influencing mortality rates in both disorders.
